# Ectopic thyroid mass in the left lateral neck and anterior mediastinum: a case report

**DOI:** 10.1186/1752-1947-8-351

**Published:** 2014-10-21

**Authors:** Jiangling Wang, Jun Fang

**Affiliations:** 1 Department of Anesthesiology, Cancer Hospital of Zhejiang Province, No. 38, Guangji Road, Hangzhou, Zhejiang Province, PR China

**Keywords:** Ectopic thyroid, Anterior mediastinum

## Abstract

**Introduction:**

Ectopic thyroid is characterized by the presence of thyroid tissue in a site other than in its usual pretracheal region. It is a rare condition among the thyroid diseases. Dural ectopic thyroid present in the cervical and anterior mediastinal has not been reported.

**Case presentation:**

A 45-year-old Chinese woman presented with a nonfunctional ectopic thyroid located both in the cervical and anterior mediastinum. The ectopic thyroid was removed under video-assisted thoracoscopic surgery using a transverse neck incision and her postoperative period has been uneventful thus far.

**Conclusions:**

Ectopic thyroid is a rare condition among the thyroid diseases, and its location in the anterior mediastinum is even more uncommon. Less than 15 cases have been reported in the last four decades. This is the first case of ectopic thyroid to appear in both the cervical and anterior mediastinum at same time. Masses in the anterior mediastinal are usually thymoma, lymphoma, pheochromocytoma and germ cell tumors. Ectopic thyroid in this area is quite rare so this case enhances our understanding of the diagnosis of mediastinal masses.

## Introduction

Ectopic anterior mediastinal thyroid is a very rare clinical entity; the incidence of this disease is less than 1 percent of all cases [1,2]. The most frequent locations are along the midline from the base of the tongue to the mediastinum [3]. Other locations like the trachea, heart, lung, duodenum, adrenal gland, gall bladder, porta hepatis, esophagus, parotid salivary gland have been reported [3-5]. Mediastinum thyroid must be differentiated from germ cell tumors, lymphomas, neurogenic tumors, thymic and mesenchymal tumors, as well as cervical goiter extending into the mediastinum and ganglioneuromas [2,6-10].

## Case presentation

**Figure 1:**
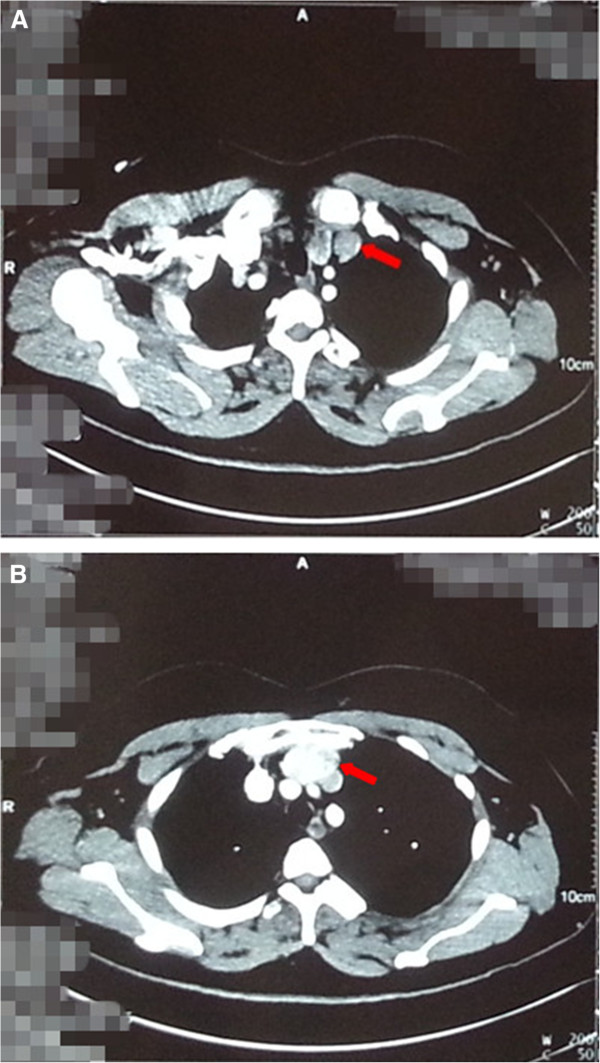
**Enhanced computed tomographic scan revealed a nodule under left thyroid lobe (Figure 1A red arrow) and an anterior mediastinum mass (Figure 1B red arrow).** Both the nodule and anterior mass have higher density as thyroid.

A 45-year-old Chinese woman was admitted to hospital because of anterior chest pain for the previous 10 days. Our patient’s height and weight were 165cm, and 75kg, respectively. Her review of systems was unremarkable and her physical examinations and laboratory tests showed no abnormalities. An enhanced computed tomography (ECT) scan revealed a 2.4×1.4×2.8cm^3^ nodule under her left thyroid lobe, mild inflammation in both lungs and a 4×2.5cm^2^ mass in her anterior mediastinum. Both the nodule and the anterior mediastinal mass displayed higher density in enhanced image, just like thyroid tissue (Figure 1).

**Figure 2:**
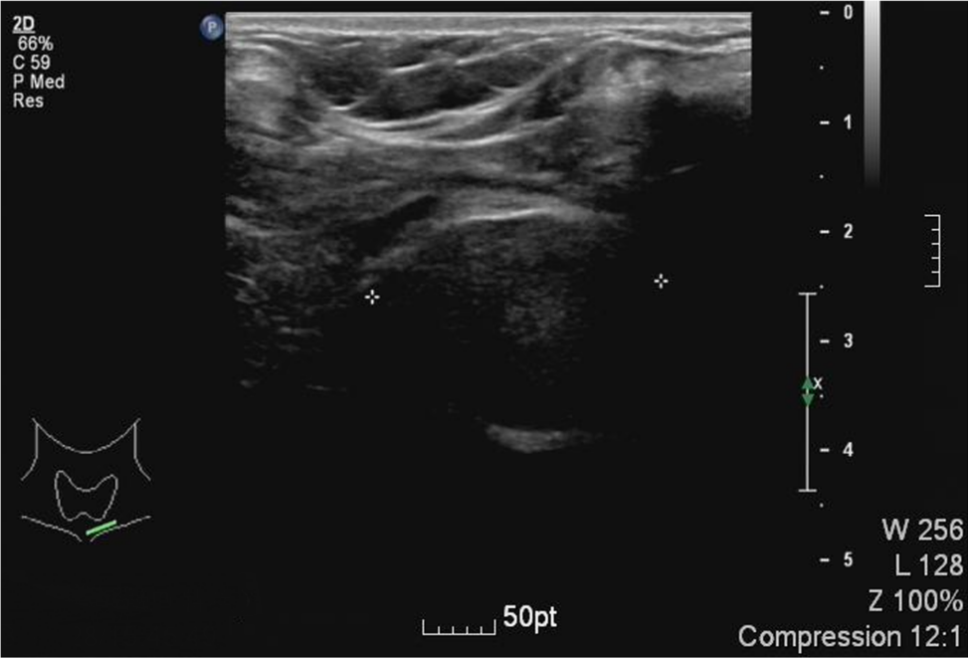
**Ultrasound scan of a separate nodule under the left thyroid lobe.** The ultrasound scan revealed a separate nodule under the left lobe.

**Figure 3:**
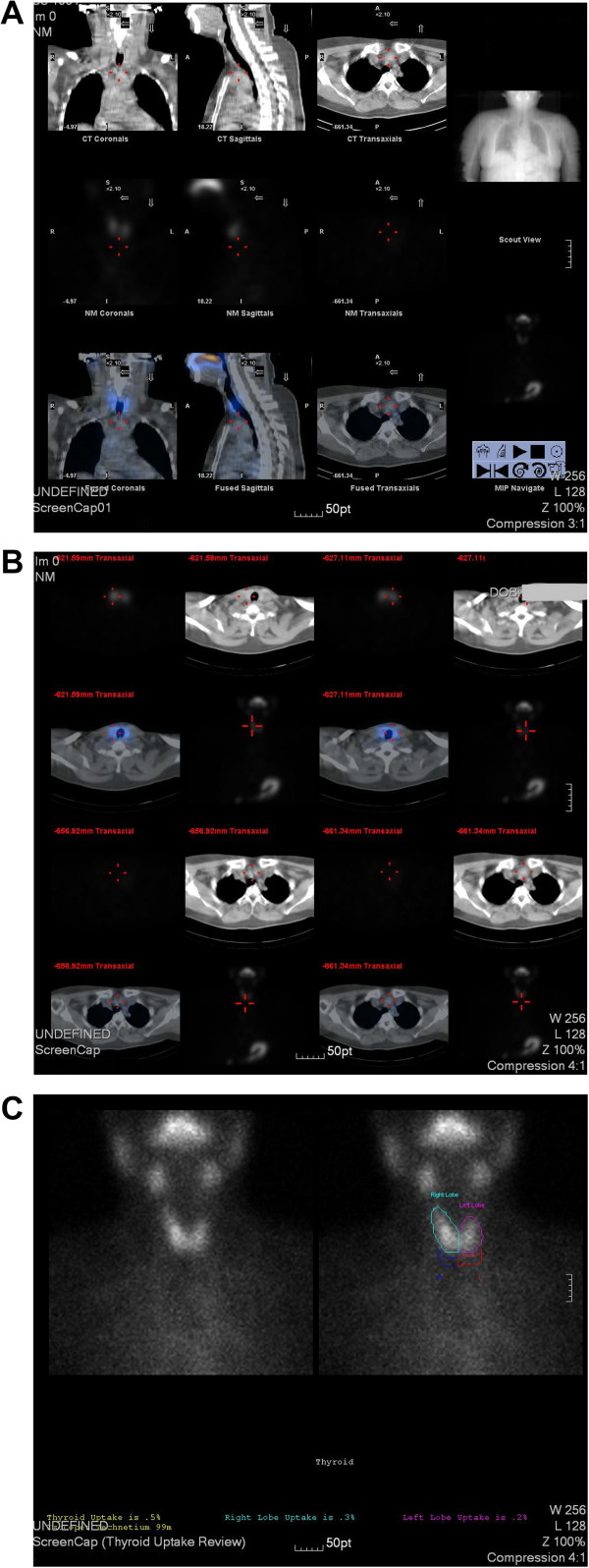
**Radioactivity uptake of the nodule and anterior mediastinal mass.** Scintigraphy, using Tc-99m, revealed both the nodule under the left thyroid lobe and the anterior mediastinal mass have no increase of radioactivity uptake in static imaging. Figure **A** revealed the coronal plane, sagittal plane and transverse plane of radioactivity uptake. Figure **B** revealed the transverse plane of radioactivity uptake and Figure **C** is the review image for the thyroid uptake.

Her blood test revealed that levels of thyroid-stimulating hormone and free thyroid hormone were normal. Antithyroglobulin antibodies and antithyroid peroxidase antibodies were also within normal range. An ultrasound of her thyroid revealed various hypoechoic nodules in her upper left lobe (the largest one measuring 0.6×0.4cm^2^) and in her lower right lobe (the largest one measuring 1.2×1.0cm^2^). Both the color Doppler flow image (CDFI) signals from these nodules were normal. There was another nodule (measuring 2.4×1.4×2.8cm^3^) under her left lobe and the CDFI signal was between level 1 to 2. No cervical adenopathy was detected and the ultrasound scan of her abdominal organs was normal (Figure 2).Technetium-99m was used to determine whether the anterior mediastinal mass was an ectopic thyroid. Compared with the higher density of radioactivity uptake in thyroid, the anterior mediastinal mass showed no increase in uptake of radioactivity in static imaging (Figure 3).

An anterior mediastinal mass resection was planned to be carried out under video-assisted thoracoscopic surgery. Total thyroidectomy was not performed as her thyroid function was normal.

The results of commonly ordered preoperative tests, including a laboratory test, pulmonary function and electrocardiogram, met with the requirements of the operation. The patient’s American Society of Anesthesiologists (ASA) physical status was class II. Advances in anesthetic technique also facilitated proper preoperative management.

**Figure 4:**
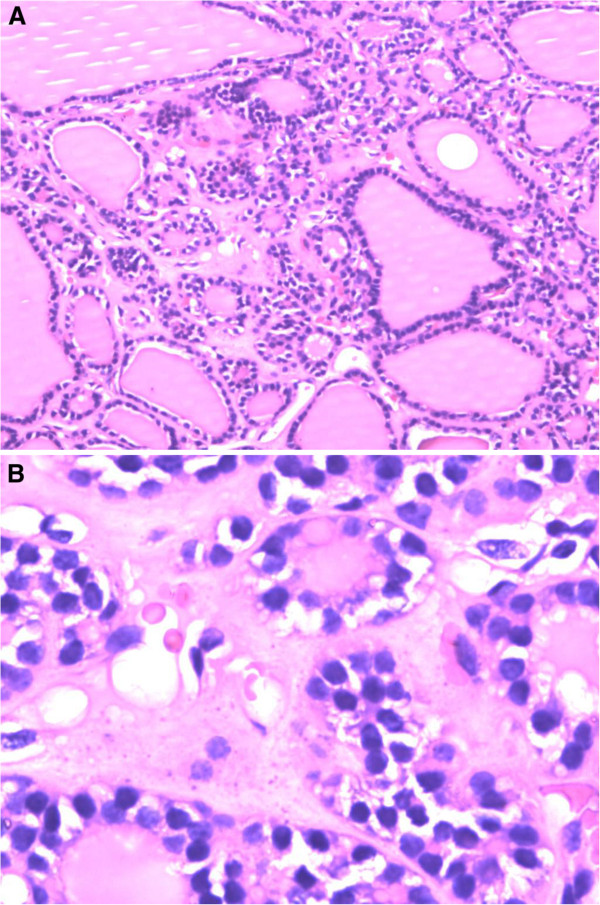
**Photomicrograph of the histopathologic examination of both the nodule and the mass.** Photomicrograph of the histopathologic examination revealed both the nodule and the mass were multinodular goiter. Hematoxylin and eosin stain. ×40 for **(A)** ×100 for **(B)**.

Our patient was placed into the right lateral recumbent (RLR) position after total intravenous anesthesia. A video thoracoscope was placed through the port in the seventh intercostal space along the left midaxillary line. Intraoperative exploration demonstrated the anterior superior mediastinum was occupied by the mass. The substernal tumor was located anteromedial to the left innominate vein, without signs of infiltration and well delineated by a capsule, measuring around 3×2cm^2^, and surrounded by blood vessels derived from intrathoracic blood vessels. The lower part of the mass was easily separated by a coagulation hook and ultrasonic knife, but the upper part was difficult to expose. A transverse neck incision was decided upon to assist the tumor excision as the nodule under her left thyroid was 0.5cm away from the lower part of the lobe, with a well-defined capsule and no obvious connection with the thyroid gland. The surgeon first removed the nodule and further intraoperative findings confirmed the encapsulated anterior mediastinum mass was behind the sternum. It had no connection to the cervical thyroid gland or to the nodule under her left lobe. The mass was separated and removed from the upper incision with thoracoscope assistance. The reports on the frozen sections of the two surgical samples were consistent with ectopic substernal thyroid tissue. A postoperative histopathologic examination of the masses, using hematoxylin and eosin, revealed both of the masses were multinodular goiters (Figure 4). Our patient had no chest pain after the mass resection. Her postoperative period was uneventful and she was discharged three days after surgery.

## Discussion

The maturation of the thyroid gland during the embryonic period follows the line from the base of the tongue to the mediastinum. Most ectopic thyroid (90 percent) have been found at the base of the tongue. Less than 1 percent has been reported in the mediastinum [1,3]. Typical substernal thyroid is always extended from the neck. Differential diagnosis for lateral cervical mass usually includes ectopia, thymic cyst and thymoma [7]. Mediastinum thyroid must be differentiated from germ cell tumors, lymphomas, neurogenic tumors and thymic and mesenchymal tumors [6]. Primary mediastinal ectopic thyroid, whose blood supply is typically from thoracic vessels, is quite rare [3,5]. Malignant transformation of ectopic thyroid is rare and is reported in about 15 percent of patients [6]. Moreover, cases of ectopic thyroid cancer are uncommon and true mediastinal thyroid cancer is extremely rare. One case was reported in 2006 [11].

Ectopic anterior mediastinal thyroid is often asymptomatic and patients are usually euthyroid. However, symptoms like cough, dyspnea, chest pain, and obstruction of the superior vena cava may occur because of the compression of organs and/or surrounding tissues. The main cause of the chest pain in this case may be due to compression of nerve(s) and tissues by the anterior mediastinal mass.

Thoracoscopy has been described as useful in both the diagnosis and resection of mediastinal masses and thoracoscopic excision has also been reported with excellent results. However, operability is unable to be confirmed earlier, not until after surgery in many cases [9].

In symptomatic cases, surgery is the treatment of choice and further treatment like radioiodine ablation and levothyroxine suppression may be needed for refractory cases located in the lateral neck [6,12]. Thoracotomy or sternotomy are mostly used for mediastinal mass excision [6]. Regular follow-up is recommended after surgery in case changes of the euthyroid multinodular goiter occur [8].

There are fewer cases reported of intrathoracic (mediastinal) thyroid and ectopic thyroid tissue developing in the thorax distinct from the cervical thyroid gland is extremely rare. Most cases of ectopic thyroid are solo nodules, unlike the case presented here where the two masses have a well-defined capsule and no obvious connection between them. Ectopic thyroid can be distinguished from secondary goiters by the following criteria: it has an independent blood supply from intrathoracic vessels rather than cervical vessels, the cervical thyroid gland is normal, the pathologic process is different between the cervical gland and the ectopic mass, and without history of malignancy [13]. This case meets all the criteria and postoperative pathology confirms it was ectopic multinodular goiter.

A thoracoscopic approach for mediastinal tumor resection is widely used because of its convenience and less trauma. A thoracoscopic approach was the first choice in this case but transverse neck incision assistance was performed due to the incomplete exposure of the anterior mass. Ectopic thyroid should be considered during the diagnosis of mediastinal masses as there is a risk of hypothyroidism after surgery if the ectopic thyroid is nonfunctional [14].

## Conclusions

In conclusion, we present an uncommon ectopic anterior mediastinal goiter with complete excision and relief of all symptoms after surgery. Although ectopic mediastinal thyroid is very rare, we should consider it in diagnosis of all mediastinal masses. Surgical resection is the first choice when there are symptoms, as they have potential to become malignant and compress surrounding tissues. Surgical excision of mediastinal ectopic thyroid is useful for both diagnosis and treatment.

## Consent

Written informed consent was obtained from the patient for publication of this case report and any accompanying images. A copy of the written consent is available for review by the Editor-in-Chief of this journal.

## Abbreviations

ASA: American Society of Anesthesiologists; CDFI: color Doppler flow image; ECT: enhanced computed tomography; RLR: right lateral recumbent.

## Competing interests

The authors declare that they have no competing interests.

## Authors’ contributions

JLW was a major contributor in writing the manuscript. JF provided important suggestions regarding medical content. Both authors read and approved the final manuscript.
